# USP49 participates in the DNA damage response by forming a positive feedback loop with p53

**DOI:** 10.1038/s41419-018-0475-3

**Published:** 2018-05-10

**Authors:** Rongfu Tu, Wenqian Kang, Xuefei Yang, Qi Zhang, Xiaoyu Xie, Wenbin Liu, Jinxiang Zhang, Xiao-Dong Zhang, Hui Wang, Run-Lei Du

**Affiliations:** 1 Hubei Key Laboratory of Cell Homeostasis, College of Life Sciences, Wuhan University, 430072 Wuhan, China; 2 College of Health Sciences and Nursing, Wuhan Polytechnic University, 430023 Wuhan, China; 3 Department of Emergency Surgery, Union Hospital, Tongji Medical College, Huazhong University of Science and Technology, 430022 Wuhan, China; 4 Department of Medical Genetics, Basic school of Tongji Medical College, Huazhong University of Science and Technology, 430030 Wuhan, China

## Abstract

The p53 tumor suppressor is a critical factor in the DNA damage response (DDR), and regulation of p53 stability has a key role in this process. In our study, we identified USP49 as a novel deubiquitinase (DUB) for p53 from a library consisting of 80 DUBs and found that USP49 has a positive effect on p53 transcriptional activity and protein stability. Investigation of the mechanism revealed that USP49 interacts with the N terminus of p53 and suppresses several types of p53 ubiquitination. Furthermore, USP49 rendered HCT116 cells more sensitive to etoposide (Eto)-induced DNA damage and was upregulated in response to several types of cell stress, including DNA damage. Remarkably, USP49 expression was regulated by p53 and USP49 in knockout mice, which are more susceptible to azoxymethane/dextran sulfate sodium (AOM/DSS)-induced colon tumors. These findings suggest that USP49 has an important role in DDR and may act as a potential tumor suppressor by forming a positive feedback loop with p53.

## Introduction

p53 is a crucial transcription factor, and its primary functions are regulating cell fate after stress and suppressing proliferation of damaged cells. Indeed, p53 is an important tumor suppresser that is mutated in more than 50% of human cancers^1–3^. The most well-characterized function of p53 is the induction of cell cycle arrest or apoptosis in response to acute DNA damage signals^3^. Because of its function in response to DNA damage, wild-type p53 is considered a guardian of the genome^4^. Under conditions of DNA damage, p53 binds to p53-responsive elements in target genes and regulates gene expression at the transcriptional level. Depending on the nature and extent of the DNA damage, different downstream genes are transcribed to initiate various cellular responses, such as cell cycle arrest, senescence, and apoptosis^5–7^. As ubiquitination of p53, which has been the focus of many studies, is a crucial posttranslational modification of the protein, the deubiquitinating enzymes (DUBs) that remove the ubiquitin moiety are also important for the activity of p53^8^.

The mammalian genome encodes ~100 DUBs that categorized into five classes: ubiquitin C-terminal hydrolases (UCHs), ubiquitin-specific proteases (USPs), ovarian tumor domain (OTU) DUBs, Machado–Joseph domain (MJD) DUBs, and a group containing a JAMM zinc metalloproteinase domain^9,10^. Early studies suggested that many DUBs, such as USP4, USP6, USP8, USP14, USP28, USP7, and UCHL5, have a prominent role in cancer development and progression^11,12^. A typical representative is USP7, which is reported to participate in various malignancies, including lung cancer^13–16^, neuroblastoma^17,18^, ovarian cancer^19,20^, breast cancer^21^, esophageal cancer^22^, colon cancer^23^, medulloblastoma^24^, glioma^25^, and leukemia^26^. Furthermore, DUBs have been implicated in numerous other pathologies such as neurological disorders, autoimmunity, inflammation, and microbial infections^27^.

Amember of the USP family, the function of USP49 is largely unknown. Nonetheless, USP49 is reported to form a complex with RuvB-like1 (RVB1) and SUG1 and to specifically deubiquitinate histone H2B. Moreover, because of its critical role in H2B ubiquitination and co-transcriptional pre-mRNA processing events, USP49 knockdown affects the abundance of isoforms expressed while only causing small changes in gene expression^28^. USP49 is also reported to suppress tumorigenesis and chemo-responses in pancreatic cancer by targeting FKBP51-AKT signaling^29^. In the present study, we screened a library consisting of 80 DUBs for novel regulators of the p53 signaling pathway. Several DUBs that modulate p53 transcriptional activity were identified, one of which was USP49. We report that USP49 binds to and stabilizes p53 via deubiquitination. In addition, p53 is essential for upregulation of USP49 mRNA and protein in response to DNA damage, which indicates that USP49 may form a positive feedback loop with p53. Finally, we found that USP49 can increase cell sensitivity to etoposide (Eto)-induced DNA damage and that USP49-knockout mice are more susceptible to colorectal cancer induced by azoxymethane/dextran sulfate sodium (AOM/DSS). These findings indicate that USP49 may act as a tumor suppressor during the genesis and development of colorectal cancer.

## Results

### Identification of candidate DUBs for p53

**Fig. 1:**
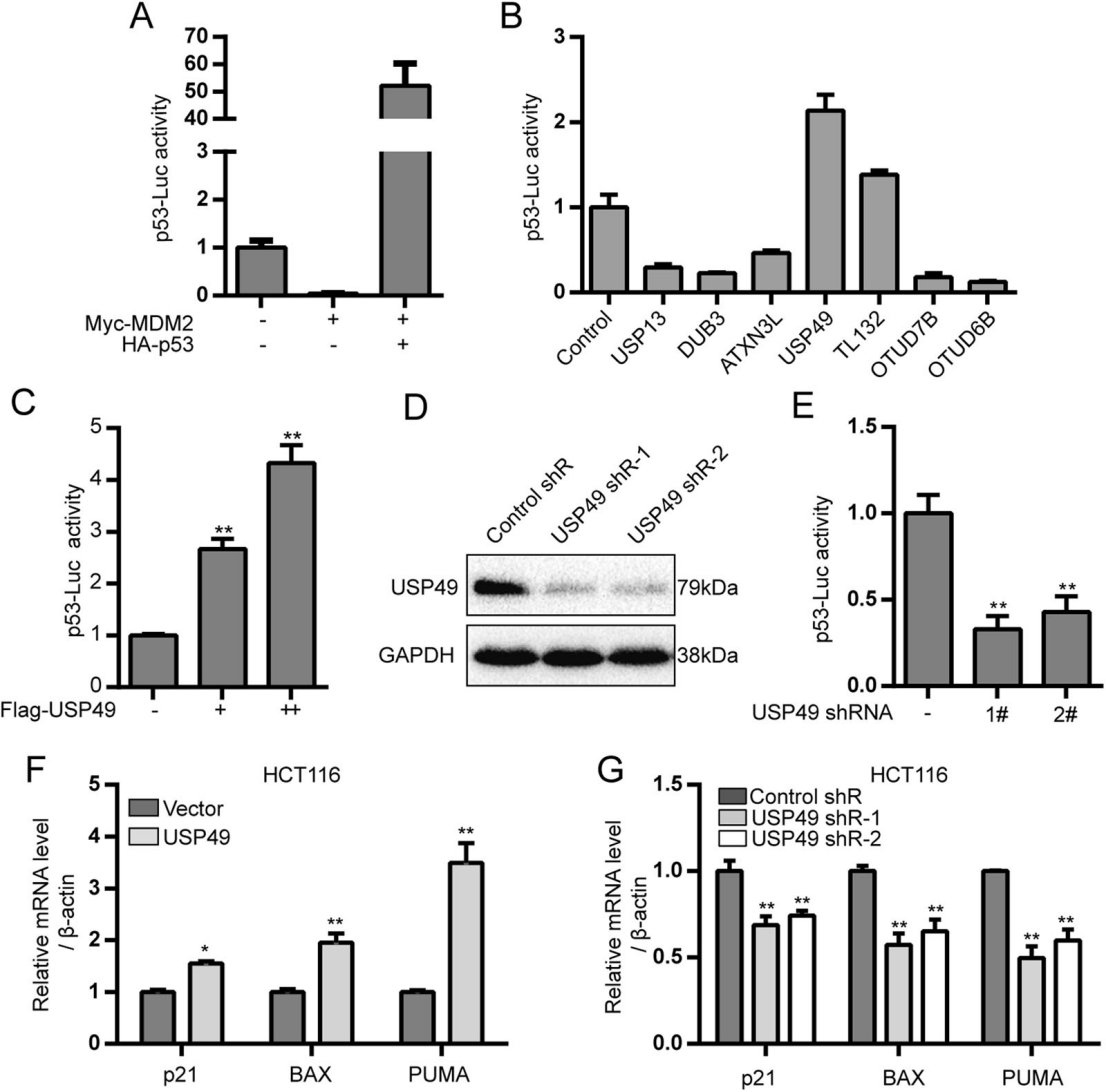
USP49 is a candidate DUB that regulates p53 transcriptional activity. **a** HEK293T cells were transfected with 50 ng of the p53-responsive reporter pp53-TA-Luc with or without 200 ng of Myc-MDM2 and 400 ng of HA-p53. Luciferase activity was measured 24 h later. **b** HEK293T cells were transfected with the p53-responsive reporter pp53-TA-Luc with or without individual DUBs. Luciferase activity was measured 24 h later. **c** HEK293T cells were transfected with the p53-responsive reporter pp53-TA-Luc with or without different doses of Flag-USP49. Luciferase activity was measured 24 h later. **d** HEK293T cells were transfected with control shRNA or USP49 shRNAs. The cells were harvested, and the USP49 protein level was measured 24 h later. **e** USP49 regulates endogenous p53 activity. HEK293T cells were co-transfected with 50 ng of pp53-TA-Luc and then transfected with 400 ng of pLKO.1 or pLKO.1-sh-USP49 plasmids. **f**, **g** Effects of USP49 on p53 targets. **f** HCT116 cells were transfected with a plasmid expressing Flag or Flag-USP49. After 24 h of incubation, total RNA was extracted. The mRNA levels of the indicated p53 targets were measured by RT-PCR and normalized to β-actin. **g** HCT116 cells were transfected with pLKO.1 or pLKO.1-sh-USP49. After 24 h of incubation, the cells were treated with 50 μM Eto for 5 h before total RNA was extracted. The mRNA levels of the indicated p53 targets were measured by RT-PCR and normalized to β-actin

Considering the complexity of the p53 regulatory network, we hypothesized that additional DUBs may be involved in the regulation of p53. To identify potential DUBs for p53, we screened a library of 80 DUBs for those that increased or decreased p53 activity. To ensure that the screening method was correct, we individually transfected Myc-MDM2 or HA-p53 into 293T cells and measured endogenous p53 transcriptional activity by a luciferase assay. As shown in Fig. 1a, p53 activity was significantly inhibited by Myc-MDM2 and notably increased by HA-p53. We then transfected individual DUBs into 293T cells and measured p53 transcriptional activity after 36 h. Next, seven candidate DUB genes were subjected to a second round of screening. As shown in Fig. 1b, two members of the OTU family, OTU6B and OTUD7B, significantly suppressed the transcriptional activity of p53. In contrast, USP49 had a profoundly positive effect on p53 activity (more than twofold). We also observed increased p53 activity when different amounts of Flag-USP49 were transfected into cells (Fig. 1c). To determine whether knockdown of endogenous USP49 has an effect on p53 activity, we designed two short hairpin RNAs (shRNAs) targeting USP49 (Fig. 1d), which resulted in reduced p53 activity (Fig. 1e). To determine whether USP49 stimulates expression of genes downstream of p53, reverse transcription-quantitative polymerase chain reaction (RT-qPCR) assays were conducted to examine the expression levels of p53 target genes. Overexpression of USP49 significantly increased p21, BAX, and PUMA mRNA levels (Fig. 1f), whereas knockdown of USP49 suppressed p21, BAX, and PUMA levels when HCT116 cells were exposed to Eto (Fig. 1g).

### USP49 upregulates p53 protein stability

**Fig. 2:**
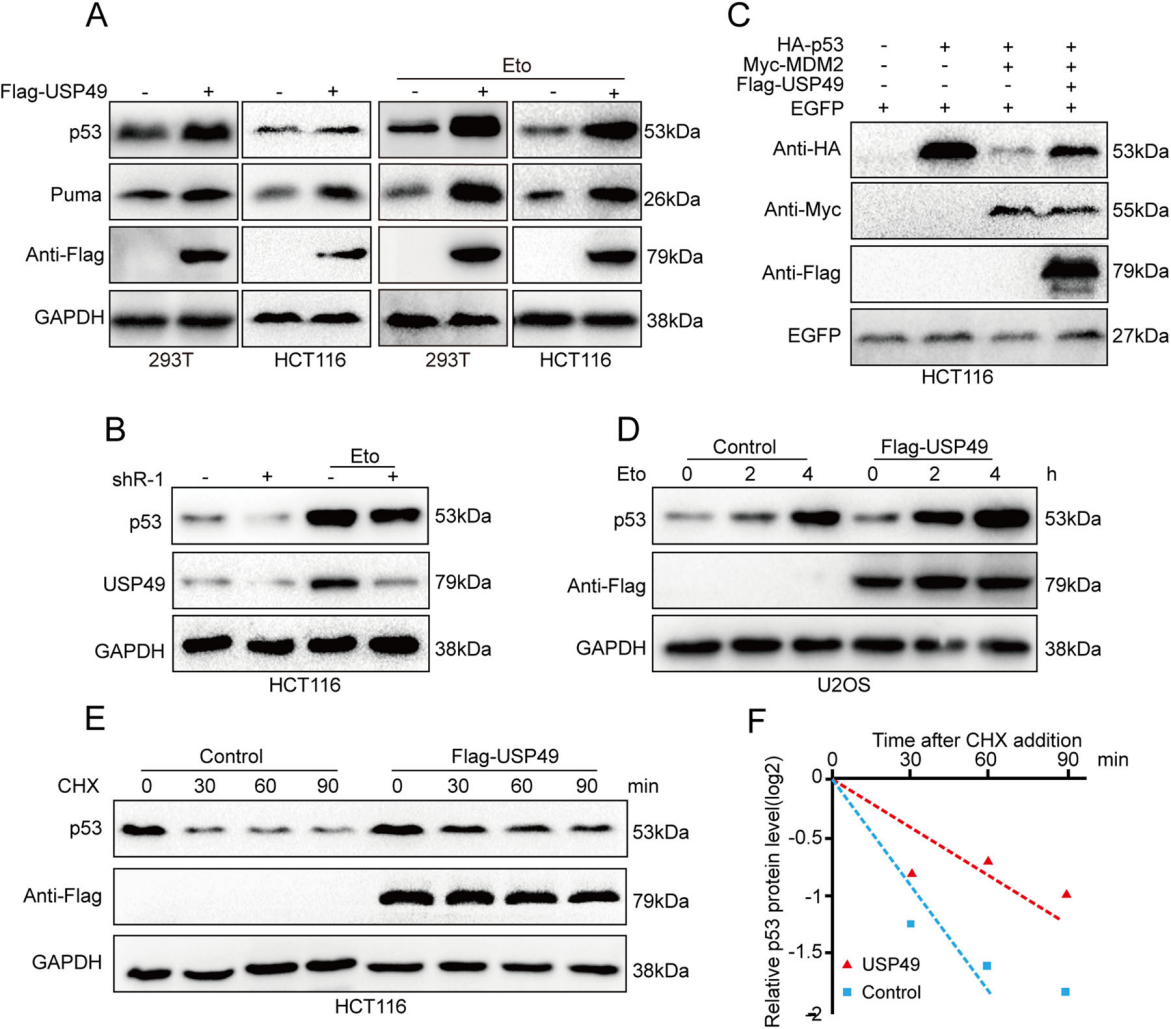
USP49 regulates p53 protein stability. **a** (Left) 293T and HCT116 cells were transfected with the vector or Flag-USP49 plasmid. After 24 h, cell lysates were analyzed by immunoblotting with the indicated antibodies. (right) 293 T and HCT116 cells were transfected with the vector or plasmid expressing Flag-USP49. After 24 h, the cells were treated with 50 μM Eto for another 4 h, and the cell lysates were analyzed by immunoblotting with the indicated antibodies. **b** HCT116 cells were transfected with pLKO.1 or pLKO.1-sh-USP49. (Left) After 24 h, cell lysates were analyzed by immunoblotting with the indicated antibodies. (Right) After 24 h of incubation, the cells were treated with 50 μM Eto for 5 h, and the cell lysates were analyzed by immunoblotting with the indicated antibodies. **c** 293T cells were transfected with the indicated plasmids. After 24 h, immunoblot analysis was performed using the indicated antibodies. **d** U2OS cells were transfected with the vector or plasmid expressing Flag-USP49. After 24 h, the cells were treated with 50 μM Eto and then harvested at the indicated times. Immunoblot analysis was performed using p53-specific antibodies. **e** HCT116 cells were infected with a lentivirus expressing Flag or Flag-USP49. The cells were treated with 50 μg/ml CHX and harvested at the indicated times. Immunoblot analysis was performed using p53-specific antibodies. **f** The bands in **e** were quantified, and the levels of p53 were normalized to those of GAPDH

To determine whether USP49 affects p53 protein levels, 293T or HCT116 cells were transfected with a Flag-USP49 plasmid, after which the protein levels of p53 and its downstream gene Puma were both upregulated with or without Eto treatment (Fig. 2a). However, knockdown of USP49 by shRNA reduced the level of p53 protein in HCT116 cells treated with or without Eto (Fig. 2b). To determine whether USP49 affects p53 degradation, we transfected HCT116 cells with p53 and MDM2 in the presence or absence of USP49 and found that USP49 drastically reduced MDM2-mediated p53 degradation (Fig. 2c). As p53 protein levels are significantly upregulated during the DNA damage response (DDR) induced by Eto, we treated U2OS cells stably transfected with or without Flag-USP49 with 50 μM Eto for various time periods to examine whether p53 is regulated by USP49 during DNA damage. Cells transfected with USP49 exhibited a large increase in p53 protein levels (Fig. 2d). We next sought to determine whether USP49 affects the half-life of the p53 protein. To this end, HCT116 cells stably transfected with or without Flag-USP49 were treated with 50 μg/ml cycloheximide (CHX) for various time points, and the results showed a longer half-life of the p53 protein in cells overexpressing USP49 (Fig. 2e, f).

### USP49 interacts with p53

**Fig. 3:**
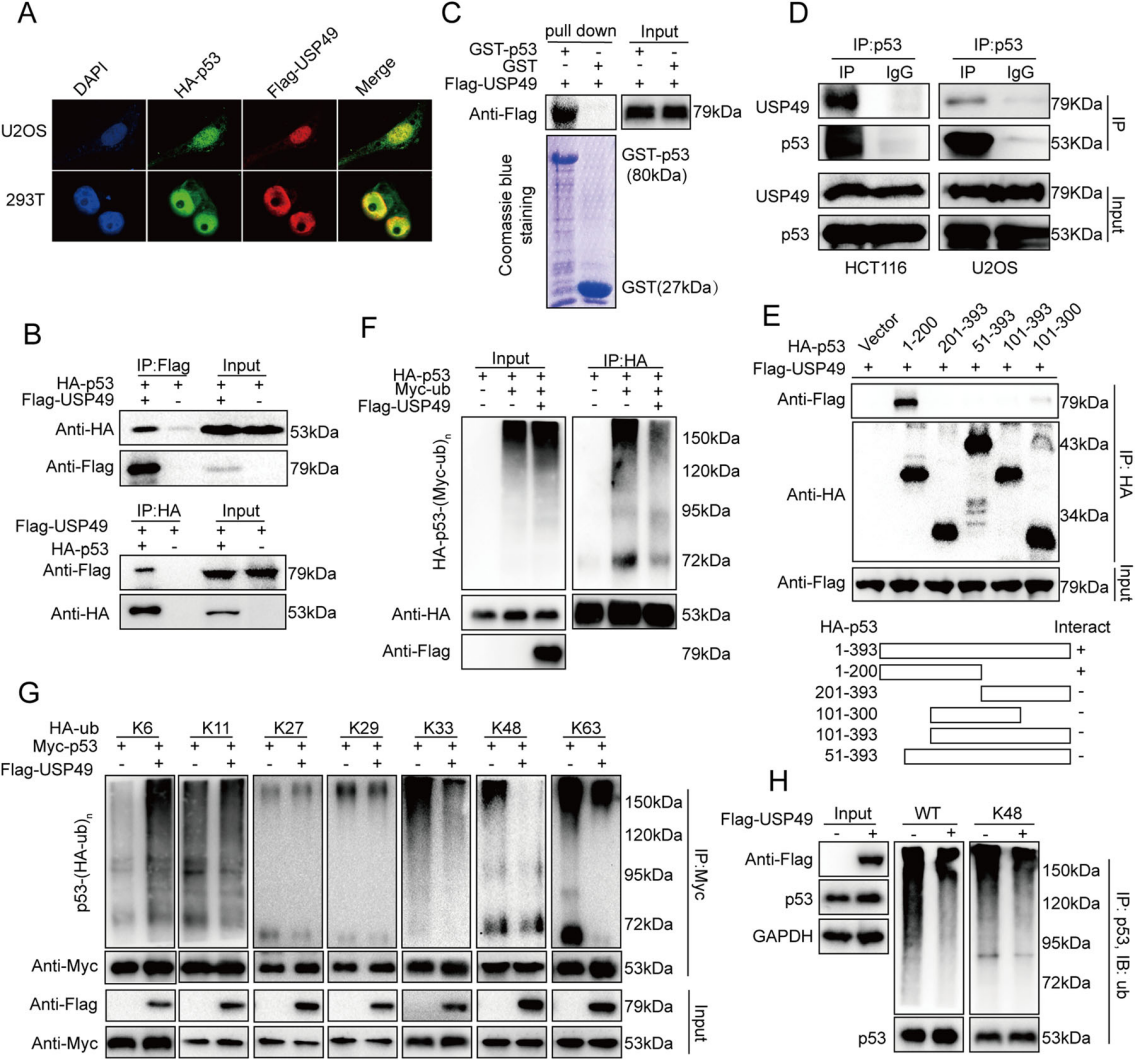
USP49 interacts with p53 and suppresses p53 ubiquitination. **a** U2OS and 293T cells were co-transfected with HA-p53 and Flag-USP49 plasmids. After 24 h, the cells were fixed with 4% paraformaldehyde, and localization of HA-p53 and Flag-USP49 was detected using an Olympus Laser Scanning Confocal Microscope. **b** HEK293T cells were co-transfected with a plasmid expressing Flag-USP49 or HA-p53 or the indicated vectors. The cell lysates were immunoprecipitated using anti-Flag or anti-HA antibodies and analyzed by immunoblotting with the indicated antibodies. **c** GST pull-down assays were performed with the indicated GST-fused proteins and cell lysates from HCT116 cells stably transfected with a plasmid expressing Flag-USP49 and analyzed by immunoblotting with the indicated antibodies. **d** HCT116 lysates were immunoprecipitated with an anti-p53 antibody; normal IgG was used as a negative control. After 24 h, the cells were treated with 50 μM Eto for another 4 h. Immunoblot analysis was performed using USP49-specific and p53-specific antibodies. **e** HEK293T cells were transfected with plasmids expressing HA-p53 or its truncations and Flag-USP49. After 24 h, the cell lysates were immunoprecipitated with an anti-Flag antibody and analyzed by immunoblotting with the indicated antibodies. **f** HEK293T cells were co-transfected with the indicated plasmids. After 24 h, the cells were subjected to denaturing immunoprecipitation using an anti-HA antibody followed by immunoblot analysis using the indicated antibodies. **g** HEK293T cells were transfected with the indicated mutant HA-ubiquitin, Myc-p53, and Flag-USP49 plasmids. After 24 h, the cells were treated with 5 μM MG132 for 12 h; the indicated types of ubiquitination were detected and quantified by denaturing immunoprecipitation and western blotting. **h** HCT116 cells stably transfected with a plasmid expressing Flag-USP49 or Flag were treated with 5 μM MG132 for 12 h. The cells were subjected to denaturing immunoprecipitation using a p53-specific antibody followed by immunoblot analysis using the indicated antibodies

We next assessed whether USP49 binds to p53. It has been reported that USP49 deubiquitinates histone H2B and regulates co-transcriptional pre-mRNA splicing; interestingly, p53 is also mainly localized to the nucleus. Confocal laser scanning microscopy showed colocalization of p53 and USP49 (Fig. 3a), raising the possibility that USP49 and p53 interact directly. We therefore performed a co-immunoprecipitation experiment in which Flag-USP49 and HA-p53 plasmids were separately transfected or co-transfected into 293T cells and potential interactions between Flag-USP49 and HA-p53 were examined (Fig. 3b). This interaction was further confirmed in a GST pull-down assay, with GST-p53 specifically interacting with transfected Flag-USP49, as shown in Fig. 3c. We also evaluated interaction between endogenous USP49 and p53 in HCT116 and U2OS cells using a p53-specific antibody (Fig. 3d). Next, we examined the binding domain preference for USP49 and p53 by transfecting Flag-USP49 and HA-p53 mutant plasmids into 293T cells and performing co-immunoprecipitation experiments using an anti-HA antibody (Fig. 3e).

### USP49 suppresses p53 ubiquitination

To explore the mechanism by which USP49 enhances p53 stability and taking into consideration that USP49 is a member of the USP family of deubiquitinating enzymes, we hypothesized that USP49 may have an effect on p53 ubiquitination. To assess this, we co-transfected HA-p53, Myc-ubiquitin, and Flag-USP49 into 293T cells, and found that USP49 suppressed ubiquitination of HA-p53 (Fig. 3f). Ubiquitin contains seven lysine (K) residues, K6, K11, K27, K29, K33, K48, and K63, through which polyubiquitin chains are linked. As the consequences of ubiquitination depend on the type of chains formed during the process^30,31^, we performed assays using seven ubiquitin mutant plasmids (only one lysine residue in each mutant) to determine which types of ubiquitination of p53 are regulated by USP49. As shown in Fig. 3g, K33-, K48-, and K63-linked ubiquitination were suppressed. For further verification, we treated HCT116 cells stably expressing Flag or Flag-USP49 with MG132, a proteasome inhibitor^32–34^, and determined the ubiquitination levels of endogenous p53. As shown in Fig. 3h, USP49 suppressed the normal and K48-linked ubiquitination of p53. Because K48-linked ubiquitination of proteins has a clear role in protein degradation by proteasomes^35^, these results support our hypothesis.

### p53 is essential for USP49 upregulation in response to DNA damage

**Fig. 4:**
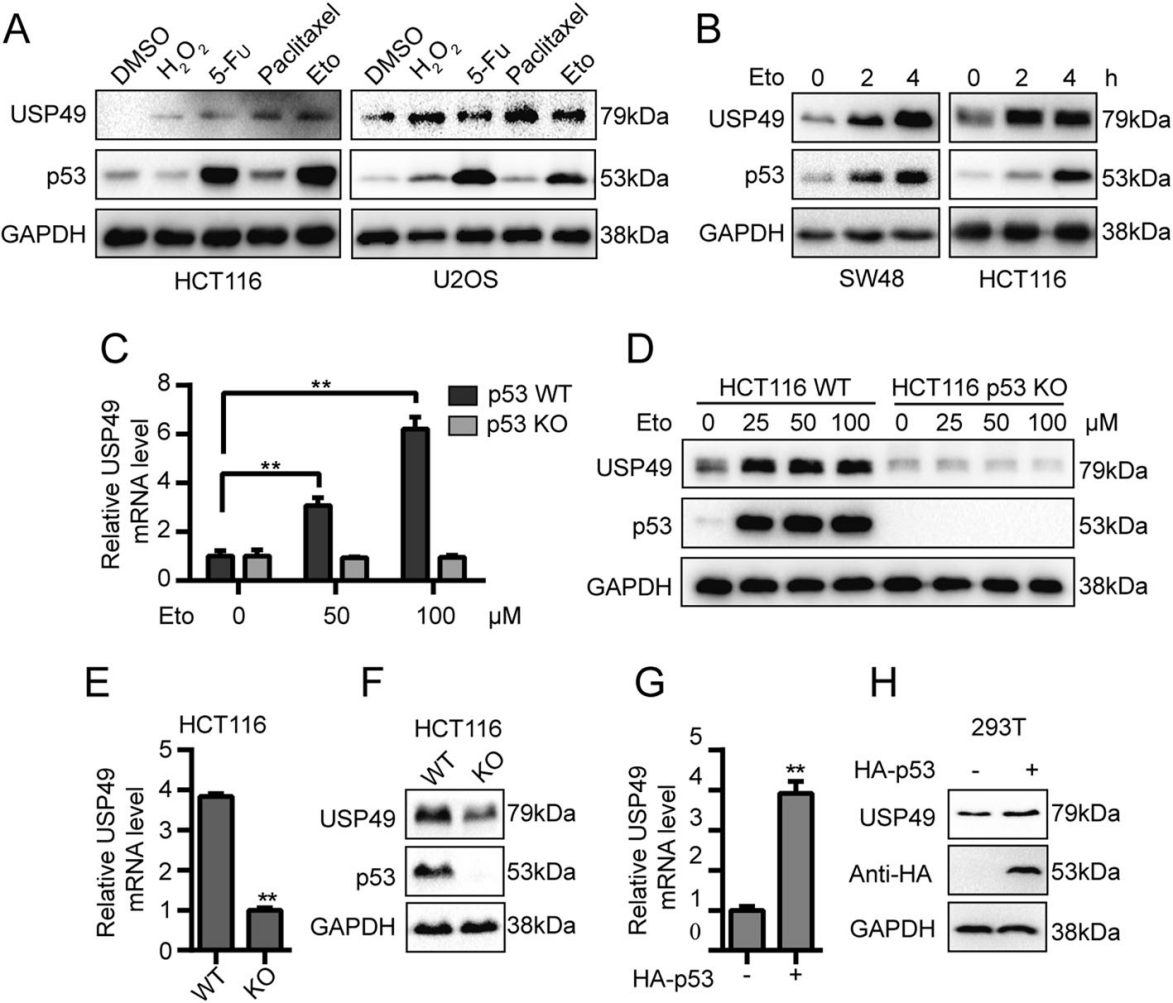
p53 is essential for USP49 upregulation in response to DNA damage. **a** HCT116 or U2OS cells were treated with 0.5 mM H_2_O_2_, 50 μM 5-Fu, 10 μM paclitaxel, or 50 μM Eto. After 6 h, the cells were harvested, and USP49 and p53 protein levels were determined by western blotting. **b** SW48 and HCT116 cells were treated with the 50 μM Eto. The cells were harvested at different time points, and immunoblot analysis was performed using USP49- and p53-specific antibodies. **c** Wild-type and p53^−/−^ HCT116 cells were treated with the indicated concentrations of Eto. After 6 h, total RNA was extracted. USP49 mRNA levels were measured by RT-PCR and normalized to β-actin levels (**p < 0.01). **d** Wild-type and p53^−/−^ HCT116 cells were treated with the indicated concentrations of Eto. After 6 h, the cells were harvested, and immunoblot analysis was performed using USP49- and p53-specific antibodies. **e** Total RNA was extracted from wild-type and p53^−/−^ HCT116 cells, and USP49 mRNA levels were measured by RT-PCR and normalized to β-actin levels (**p < 0.01). **f** Wild-type and p53^−/−^ HCT116 cell lysates were subjected to immunoblot analysis with USP49- and p53-specific antibodies. **g** HEK293T cells were transfected with or without the HA-p53 plasmid. After 24 h, total RNA was extracted. USP49 mRNA levels were measured by RT-PCR and normalized to β-actin levels (**p < 0.01). **h** HEK293T cells were transfected with or without the HA-p53 plasmid. After 24 h, the cell lysates were analyzed by immunoblotting with the indicated antibodies

Eto is an anticancer drug that functions as an inhibitor of Topoisomerase II, an enzyme that is essential for DNA replication, chromosome condensation and chromosome segregation^36,37^. In vitro studies have shown that Eto increases Topoisomerase II-mediated DNA breakage primarily by inhibiting the ability of the enzyme to religate cleaved nucleic acid molecules^38^. p53 has a key role in DDR, and USP49 was found in our study to regulate p53 activity. Thus, to determine whether USP49 protein levels are altered when cells are stressed, HCT116 and U2OS cells were treated with various concentrations of H_2_O_2_, fluorouracil (5-Fu), paclitaxel, or Eto. USP49 was induced by all four reagents (Fig. 4a). We next treated SW48 and HCT116 cells for various durations and found that USP49 was induced during early stages (Fig. 4b). Furthermore, USP49 mRNA was upregulated in HCT116 wild-type cells, but not in HCT116 p53^−/−^ cells, in response to Eto (Fig. 4c), which suggested that the increase in USP49 depended on p53. To verify this, USP49 protein levels were detected in Eto-treated wild-type and p53^−/−^ HCT116 cells, and p53 was essential for USP49 upregulation (Fig. 4d).

Interestingly, p53 was also found to regulate USP49. As shown in Fig. 4e and f, both the mRNA and protein levels of USP49 were downregulated in HCT116 p53^−/−^ cells. Furthermore, when 293T cells were transfected with the HA-p53 plasmid, mRNA and protein levels of USP49 in cells were upregulated (Fig. 4g, h). These results indicate that USP49 may form a positive feedback loop with p53.

### USP49 deletion renders cells more resistant to DNA damage and promotes tumor genesis in a murine model

**Fig. 5:**
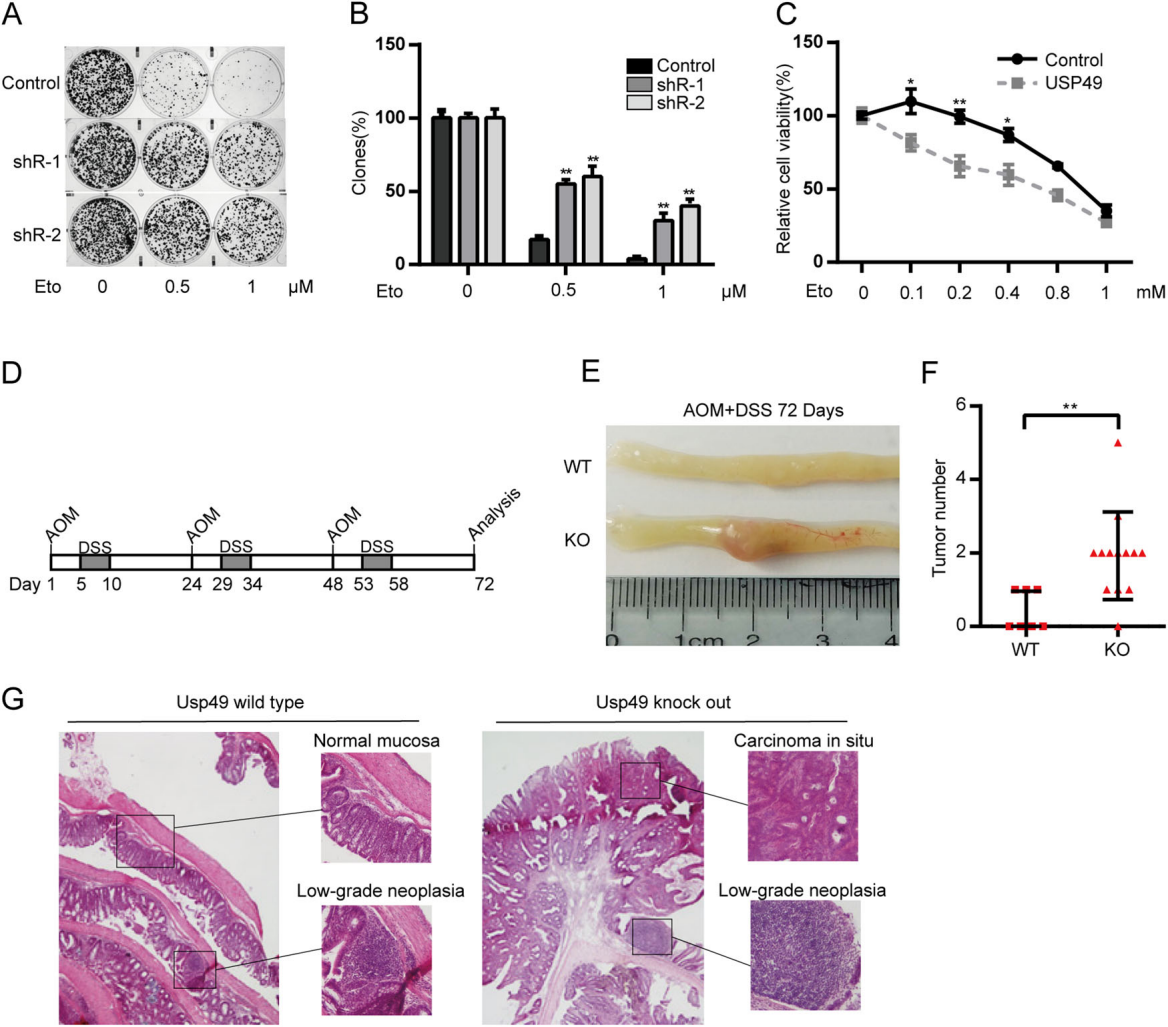
USP49 deletion renders cells more resistant to DNA damage and promotes tumor genesis in a murine model. **a** A total of 2000 HCT116 cells stably expressing shRNAs targeting USP49 or control shRNAs were seeded in six-well plates with or without the indicated concentrations of Eto. After 24 h, the medium was replaced with fresh McCoy’s 5A medium. Approximately 10 days later, the clones were stained with crystal violet, and clone formation was analyzed (*p < 0.05, **p < 0.01). **b** The numbers cell clones in **a** were quantified, and the levels of different cell lines were normalized to those of their control groups. **c** HCT116 cells stably expressing Flag-USP49 or a control plasmid were seeded in 96-well plates; 24 h later, the cells were treated with the indicated concentrations of Eto for another 24 h. Cell viability was evaluated using the CCK-8 assay. Significant differences between groups are shown (**p < 0.01). **d** Schematic representation of AOM and DSS treatment. **e** Animals were treated as described in **d** and killed, and colorectal samples were collected for imaging. **f** Graph showing colon tumor numbers at week 10. **g** Representative H&E-stained histological images at week 10

HCT116 cells stably transfected with shRNAs targeting USP49 were exposed to Eto. As shown in Fig. 5a and b, knockdown and overexpression of USP49 increased cell resistance or sensitivity to DNA damage, respectively (Fig. 5c). To examine the role of USP49 in tumor genesis, USP49-knockout mice were used to produce an AOM/DSS-induced colorectal cancer model (Fig. 5d). Mice were killed at the end of the protocol, and their colons were resected to assess the incidence and number of tumors. The tumor incidence in USP49-knockout mice was >90%, but it was <50% in USP49-wild-type mice. Moreover, compared to the USP49-wild-type mice, the USP49-knockout mice displayed a notable increase in the number of macroscopically visible tumors (Fig. 5e, f) as well as larger and more advanced tumors and an increased incidence of high-grade intraepithelial neoplasia and carcinoma in situ (Fig. 5g).

## Discussion

Under physiological conditions, the p53 protein is maintained at low levels through ubiquitin-mediated degradation, with p53 activity being regulated by more than 15 ubiquitin ligases^39^. The most important E3 ligase of p53 is MDM2, a direct transcriptional target of p53 that has a major role in regulating p53 protein stability^40–42^. In this feedback loop, p53 binds to the promoter of MDM2 and stimulates its transcription; MDM2 can also bind to p53 and mediate multiple mono-ubiquitination events, promoting nuclear export and degradation of p53^39^. Ubiquitination is a reversible process, and several DUBs are reported to regulate p53 protein stability. The role of HAUSP in the p53 pathway appears to be unique: on the one hand, overexpression of HAUSP stabilizes both p53 and MDM2 and, more importantly, activates p53; on the other hand, HAUSP ablation destabilizes MDM2 and activates p53^43,44^. It has also been reported that DNA damage caused USP10 stabilization and nuclear translocation of a subset of these molecules to activate p53^45^. In contrast, USP4 and USP5 are reportedly involved in suppressing p53^8,46^.

USP49 might regulate nucleosome stability by altering the ubiquitination level of H2B, and USP49 has a critical role in co-transcriptional pre-mRNA processing events^28^. In our study, we found that USP49 has a positive effect on the transcriptional activity and protein stability of p53 and that USP49 interacts with p53 and suppresses its ubiquitination. Furthermore, USP49 was activated at both the mRNA and protein levels in response to DNA damage, though this activation was not observed in p53^−/−^ cells. Finally, USP49 was shown to improve cell sensitivity to DNA damage signals. These results indicate that USP49 is an interesting downstream target of p53 that regulates its activity. Several limitations of our study should be considered. As depicted in Fig. 4a, USP49 protein levels were significantly upregulated upon stimulation with paclitaxel, whereas no apparent increase in p53 levels occurred. We have no explanation for these results. Another limitation of our study is that the specific mechanism by which USP49 is regulated by p53 was not clearly defined. We will investigate these issues in future studies.

**Fig. 6:**
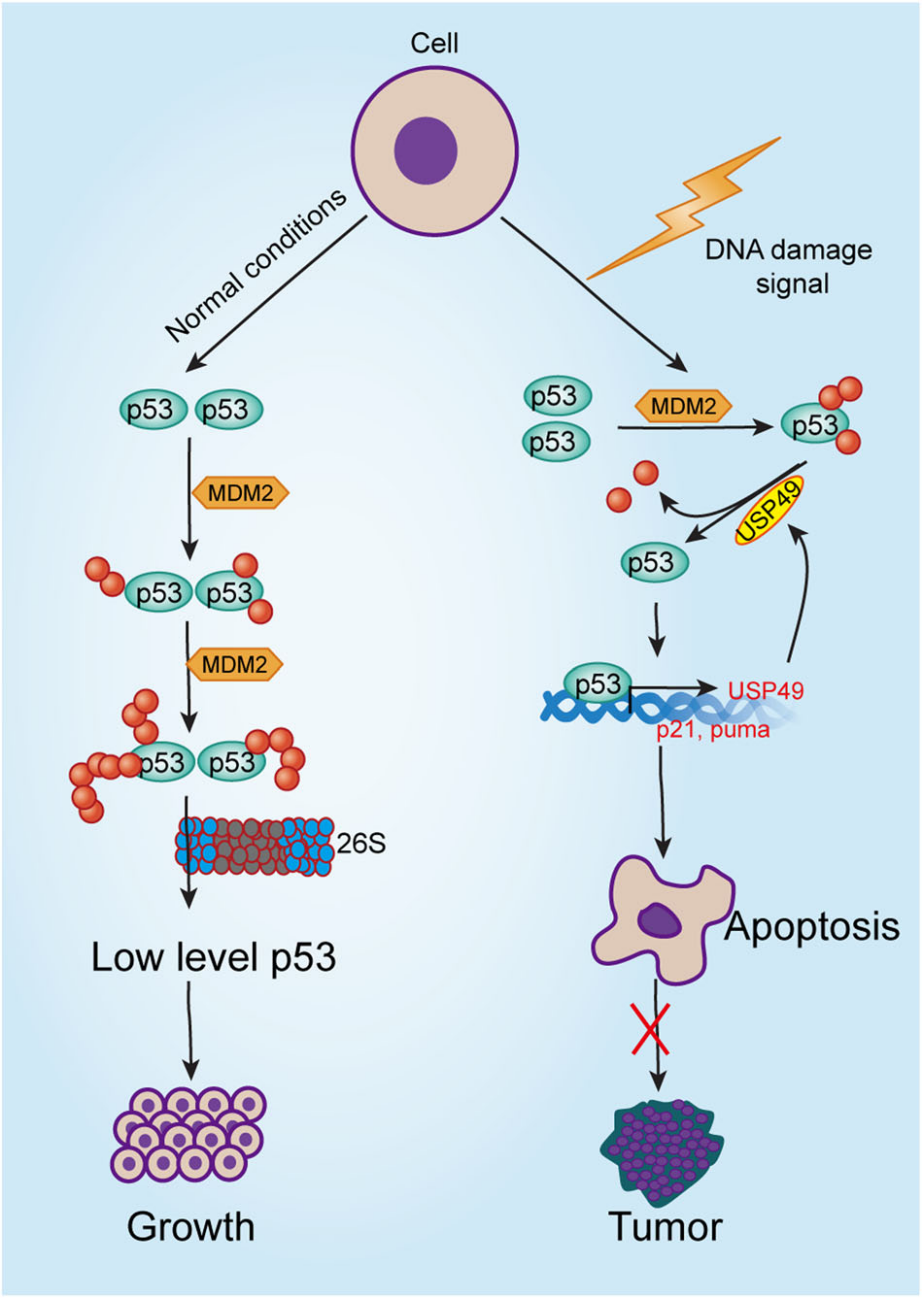
Model of positive feedback between USP49 and p53. **a** Under normal conditions, the p53 protein is maintained at low levels by MDM2-mediated ubiquitination and degradation. Under conditions of DNA damage, USP49 suppresses p53 ubiquitination and promotes p53 stabilization. Furthermore, p53 upregulates USP49 mRNA and protein levels in response to DNA damage

In conclusion, we demonstrate that USP49 acts as an important regulator of DNA damage and tumorigenesis by forming a positive feedback loop with p53 (Fig. 6). Our findings suggest that USP49 may be a potential target for cancer therapy.

## Materials and methods

### Cell culture

Human HCT116 wild-type and HCT116 p53^−/−^ cells were cultured in McCoy’s 5A medium (AppliChem, Darmstadt, Germany) supplemented with 10% fetal bovine serum (FBS, HyClone, Logan, UT, USA) and 100 U of penicillin–streptomycin (Gibco, Carlsbad, CA, USA) at 37 °C in a 5% CO_2_ incubator. HEK293T, U2OS, and SW48 cells were cultured in Dulbecco’s modified Eagle’s high-glucose medium (HyClone) with 10% FBS and 100 U of penicillin–streptomycin in a 5% CO_2_ incubator.

### Antibodies and reagents

An anti-rabbit monoclonal antibody against USP49 (cat# A3402, Abclonal, USA), anti-mouse monoclonal antibody against p53 (Santa Cruz Biotechnology, USA), anti-rabbit monoclonal antibody against Puma (cat# 4076P, CST, USA), and anti-mouse monoclonal antibody against GAPDH (cat# CW0266A, Beijing Cowin Biotech, China) were used. Anti-mouse monoclonal anti-HA tag (cat# M180-3), anti-Flag tag (cat# M185-3L), and anti-Myc tag (cat# M192-3) antibodies were purchased from Medical and Biological Laboratories (MBL, Nagano, Japan). Glutathione Sepharose 4B (cat# 17075601) was purchased from GE Healthcare Bio-Sciences AB (Shanghai, China). The proteasome inhibitor MG132 (Selleckchem, cat# S2619), Eto (Selleckchem, cat# S1225), and CHX (Sigma, cat# C7698) were purchased from the indicated manufacturers.

### Plasmids

Plasmids expressing Flag-USP49 were generated by polymerase chain reaction (PCR) and cloned into phage-6 tag. HA-p53 and its mutated forms were cloned into PCDNA5/FRT/TO-Flag/HA. Myc-MDM2 was generated by PCR and cloned into pCMV-3Tag-2A. GST-p53 was generated by PCR and cloned into pGEX 6p-1. Myc-ubiquitin was kindly provided by Hong-Bing Shu (Wuhan University, Wuhan, China). HA-ubiquitin mutants were kindly provided by Bo Zhong (Wuhan University, Wuhan, China). pGL3-TK-luc and pGL3-p53-luc were purchased from Addgene (Cambridge, MA, USA). GST-p53 was generated by PCR and cloned into pGEX 6p-1. The sequences of shRNAs used to target USP49 were as follows: USP49 shR-1 forward, 5′-CC GGCTCAGTCAGGTCACATGTATACTCGAGTATACA TGTGACCTGACTGAGTTTTTG-3′; USP49 shR-1 reverse, 5′-AATTCAAAAA CTCAGTCAGGTCACATGTATACTCGAGTATACATGTGACCTGACTGAG-3′; USP49 shR-2 forward, 5′- CCGGGCCGTAATCATCGAGAGAAGACTCGAGTCT TCTCTCGATGATTACGGCTTTTTG-3′; and USP49 shR-2 reverse, 5′-AATT CAAAAAGCCGTAATCATCGAGAGAAGACTCGAGTCTTCTCTCGATGATTACGGC-3′.

### Transfection

Transient transfections were performed using Lipofectamine 2000 (Invitrogen, Grand Island, NY, USA) as described previously according to the manufacturer’s instructions (Life Technologies, Inc., Grand Island, NY, USA). Transfection reagents and DNA were mixed in Opti-MEM (Invitrogen) and added to cells grown to 60–80% confluence; the medium was replaced with fresh medium after 4–6 h.

### Reporter assay

The reporter assay was performed as previously described. HEK293T or HCT116 cells were co-transfected with pGL3-p53-luc (for p53 activation), pGL3-TK-luc and the indicated amounts of expression constructs using Lipofectamine 2000 according to the manufacturer’s instructions. Luciferase activity was determined using the luciferase assay system and chemiluminescent reagents from Promega (Madison, WI, USA).

### RNA quantification

RNA quantification was performed as previously described. Total RNA was extracted using TRIzol reagent (Takara Biotechnology, Dalian, China) according to the manufacturer’s protocol, and RNA concentrations were determined using a NanoDrop instrument (NanoDrop Technologies). Complementary DNA was prepared using a First-Strand cDNA Synthesis Kit (Roche Diagnostics, Mannheim, Germany). The following primers were used for qPCR: USP49 forward, 5′-AGTTTGGGAGTTC CCTCCTT-3′; USP49 reverse, 5′-GCTGCTCTCCTGTGTGGATA-3′; BAX forward, 5′-ACTCCCCCCGAGAGGTCTT-3′; BAX reverse, 5′-GCAAAGTAGAAAAGGGCGACAA-3′; p21 forward, 5′-CTGGACTGTTTTCTCTCGGCTC-3′; p21 reverse, 5′-TGTATATTCAGCATTGTGGGAGGA-3′; PUMA forward, 5′-GACCTCAACGCACAGTACGAG-3′; PUMA reverse, 5′- AGGAGTCCCATGATGAGATTGT-3′; β-actin forward, 5′-GACAGCAGTTGGTTGGAG-3′; and β-actin reverse, 5′-GGGT GAGGGACTTCCTGTAA-3′.

### Generation of stable cell lines

Stable cell lines were generated by lentivirus infection. HEK293T cells were co-transfected with the indicated plasmids and lentiviral packaging vectors; after 48 h, viral supernatants were filtered through a 0.45-μm bacterial filter and added to HCT116 cells or other cell types. The infected cells were selected by puromycin treatment for at least 48 h before additional experiments were performed.

### Immunoblot analysis

Immunoblot analysis was performed as described previously. Cells were lysed in RIPA buffer (50 mM Tris-HCl pH 7.4, 150 mM NaCl, 1% v/v NP-40, 1 mM ethylenediaminetetraacetic acid (EDTA), and 0.1% w/v sodium dodecyl sulfate (SDS)) and then centrifuged at 13,000×*g* for 10 min at 4 °C. Protein concentrations were determined using Pierce® BCA Protein Assay Kit (Pierce, 23225). Equal amounts of protein (30–150 µg) were separated by 10% SDS-polyacrylamide gel electrophoresis (PAGE) and transferred to polyvinylidene fluoride (PVDF) membranes (Millipore, cat# IPVH00010, Merck KgaA, Darmstadt, Germany). The membranes were blocked with 5% non-fat dry milk in TBST (20 mM Tris-HCl, pH 7.5, 150 mM NaCl, and 0.1% Tween-20) for 1 h at room temperature, incubated with the primary antibody on a rocking platform overnight at 4 °C, and then incubated with a horseradish peroxidase-conjugated secondary antibody. Protein bands were visualized using the SuperSignal chemiluminescence kit (Merck Millipore), and the Bio-Rad ChemiDoc XRS + imaging system (Bio-Rad, USA) was used for signal detection.

### Immunoprecipitation

The immunoprecipitation assay was performed as previously described. Cells were lysed in lysis buffer (50 mM Tris-HCl, pH 7.4–7.5, 150 mM NaCl, 1 mM EDTA, 1% NP-40, 5 μg/ml aprotinin, and 5 μg/ml leupeptin). Cell lysates were incubated with the indicated antibodies and protein G-agarose beads (Roche Ltd.) at 4 °C for 4 h. The beads were washed three times with 1 ml of lysis buffer. The precipitates were subjected to SDS-PAGE, and subsequent immunoblot analysis was performed using the indicated antibodies.

### GST pull-down assay

Expression and purification of GST and the GST-p53 fusion protein were performed as previously described. Flag-USP49 proteins, obtained from whole-cell lysates of HCT116 cells stably expressing Flag-USP49, were incubated with GST and GST-p53 fusion protein bound to Sepharose beads in 1 ml of RIPA buffer at 4 °C for 4 h. The beads were then washed and analyzed by immunoblotting.

### Immunostaining

Immunostaining was performed as previously described. U2OS and 293T cells were cultured in 12-well plates with coverslips (cat# 801007; NEST, Wuxi, China) and fixed with 4% paraformaldehyde (diluted in phosphate-buffered saline (PBS)) for 15 min. The cells were then washed three times with PBS and permeabilized in 0.5% Triton X-100 in PBS for 10 min. The cells were then washed with PBS and blocked with 3% bovine serum albumin (BSA) in PBS for 1 h. Mouse anti-Flag and rabbit anti-HA primary antibodies were used, and mouse Alexa Fluor 594-conjugated and rabbit Alexa Fluor 488-conjugated antibodies (Invitrogen) were used as secondary antibodies. DAPI (4,6-diamidino-2-phe-nylindole) was used to stain the nucleus. Immunostained cells were visualized and photographed using an Olympus Laser Scanning Confocal Microscope.

### Induction of colitis-associated colorectal cancer in mice

Animal experiments were performed after approval by the National Resource Center for Mutant Mice (NRCMM). Eight-week-old, female, C57BL/6 mice were injected with 10 mg/kg of AOM intraperitoneally at the beginning of the experiment. After 4 days, 2% (w/v) DSS (molecular weight: 36,000–50,000) was given orally in drinking water over 5 days, after which normal water was given during a 14-day period of recovery. This cycle was repeated three times, and the mice were killed 2 weeks after the last DSS cycle.

### H&E staining

Sections from the rectum of the mice were exposed to formalin for 24 h, subjected to 30% sugar dehydration for 36 h, embedded with suitable reagents, and used to produce frozen samples. The samples were sectioned and stained according to a standard protocol. After staining, photographs were obtained by optical microscopy.
